# Antimicrobial peptide A20L: *in vitro* and *in vivo* antibacterial and antibiofilm activity against carbapenem-resistant *Klebsiella pneumoniae*

**DOI:** 10.1128/spectrum.03979-23

**Published:** 2024-07-09

**Authors:** Huijing Zhou, Xin Du, Yue Wang, Jingchun Kong, Xiaodong Zhang, Weixiang Wang, Yao Sun, Cui Zhou, Tieli Zhou, Jianzhong Ye

**Affiliations:** 1 Department of Clinical Laboratory, The First Affiliated Hospital of Wenzhou Medical University; Key Laboratory of Clinical Laboratory Diagnosis and Translational Research of Zhejiang Province, Wenzhou, Zhejiang, China; 2 Department of Medical Lab Science, School of Laboratory Medicine and Life Science, Wenzhou Medical University, Wenzhou, Zhejiang, China; The University of North Carolina at Chapel Hill, Chapel Hill, North Carolina, USA

**Keywords:** antimicrobial peptide, A20L, *Klebsiella pneumoniae*, carbapenem-resistance, antibacterial, antibiofilm

## Abstract

Antimicrobial resistance has become a growing public health threat in recent years. *Klebsiella pneumoniae* is one of the priority pathogens listed by the World Health Organization. Antimicrobial peptides are considered promising alternatives to antibiotics due to their broad-spectrum antibacterial activity and low resistance. In this study, we investigated the antibacterial activity of antimicrobial peptide A20L against *K. pneumoniae. In vitro* antibacterial activity of A20L against *K. pneumoniae* was demonstrated by broth microdilution method. We confirmed the *in vivo* efficacy of A20L by *Galleria mellonella* infection model. In addition, we found that A20L also had certain antibiofilm activity by crystal violet staining. We also evaluated the safety and stability of A20L, and the results revealed that at a concentration of ≤128 µg/mL, A20L exhibited negligible toxicity to RAW264.7 cells and no substantial toxicity to *G. mellonella*. A20L was stable at different temperatures and with low concentration of serum [5% fetal bovine serum (FBS)]; however, Ca^2+^, Mg^2+^, and high serum concentrations reduced the antibacterial activity of A20L. Scanning electron microscope (SEM) and membrane permeability tests revealed that A20L may exhibit antibacterial action by damaging bacterial cell membranes and increasing the permeability of outer membrane. Taken together, our results suggest that A20L has significant development potential as a therapeutic antibiotic alternative, which provides ideas for the treatment of *K. pneumoniae* infection.

**IMPORTANCE:**

A20L showed antibacterial and anti-infective efficacy *in vitro* and *in vivo* against *Klebsiella pneumoniae*. It can have an antibacterial effect by disrupting the integrity of cell membranes. A20L displayed anti-biofilm and anti-inflammatory activity against carbapenem-resistant *K. pneumoniae* and certain application potential *in vivo*, which provides a new idea for the clinical treatment of biofilm-associated infections.

## INTRODUCTION

Antimicrobial resistance (AMR) is one of the world’s most severe public health problems due to the increasing number of drug-resistant pathogens encountered by health workers (1). AMR is associated with an increased risk of treatment failure and recurrent infections, imposing health and economic burdens (2). *Klebsiella pneumoniae* is a gram-negative and capsulated bacteria, prevalent in the environment that is predominantly associated with community-acquired pneumonia (3, 4). In addition to pneumonia, *K. pneumoniae* can also cause urinary and lower biliary tract infections, surgical wound site infections, and bloodstream-related infections (3, 5). Carbapenems are used as defensive drugs to treat *K. pneumoniae* infections (6). Overuse of antibiotics has resulted in the emergence of carbapenem-resistant *Klebsiella pneumoniae* (CRKP) (7–9). These *K. pneumoniae* strains are associated with significant mortality rates, particularly in critically sick and immunocompromised patients (10). As a result, alternative anti-infection strategies are desperately needed.

Antimicrobial peptides (AMPs) are small molecular peptides with 12–50 amino acid residues (11–13). AMPs possess the advantages of high solubility, small molecular weight, and low resistance development (14). Because of these benefits, AMPs have potential applications in medicine, and some AMPs are now in clinical trials as antibacterial agents (15). Researchers have traditionally sought for AMPs with low toxicity and high activity (16).

A20L is a derivative of AMP V13K, in which the amino acid alanine at site 20 is replaced by leucine (17). Studies have shown that A20L has antibacterial activity against *Escherichia coli*, *Pseudomonas aeruginosa*, *Staphlococcus aureus*, and *Bacillus subtilis* (17). They have only been preliminarily investigated for their antibacterial activity through drug sensitivity experiments. At present, there is no detailed report on the therapeutic effect of this AMP on *K. pneumoniae*. Here, we further investigated the antibacterial activity of A20L against clinical *K. pneumoniae* isolates *in vitro* and *in vivo* and the underlying mechanisms. The antibiofilm activity of A20L was also investigated. In addition, we evaluated the stability and safety of the peptide under different conditions. Our results suggested that A20L is a potential antibiotic alternative against *K. pneumoniae* and is worthy of further clinical investigation.

## RESULTS

### Physicochemical properties and structural prediction of A20L

The structure and physicochemical properties of A20L were predicted by Expasy, Prabi, and Phyre2 software. Prabi software prediction results showed that the molecular weight of AMP A20L is 2,991.66, with 26 amino acid residues, carrying 7 positive charges, isoelectric point is 10.78, fat solubility index is 93.85, and hydrophilicity index is −0.138 (GRAVY positive value represents hydrophobicity, negative value represents hydrophilicity, which is used to evaluate the solubility of AMP). The results showed that A20L had good water solubility. The instability index is 6.56 (instability index <40 indicates that AMP is stable), which indicates that A20L has good stability (Table 1).

**TABLE 1:** Physicochemical properties of AMP A20L^*a*^

Peptide	Molecular weight	Residue	Net charge	PI	Aliphatic index	GRAVY	Instability index	Alpha helix
A20L	2,991.66	26	7	10.78	93.85	−0.138	6.56	96.15

^*a*^ Positive value of GRAVY represents hydrophobicity, and negative value represents hydrophilicity, which is used to evaluate the solubility of AMPs. Instability index <40 indicates that AMPs are stable.

**Fig 1:**
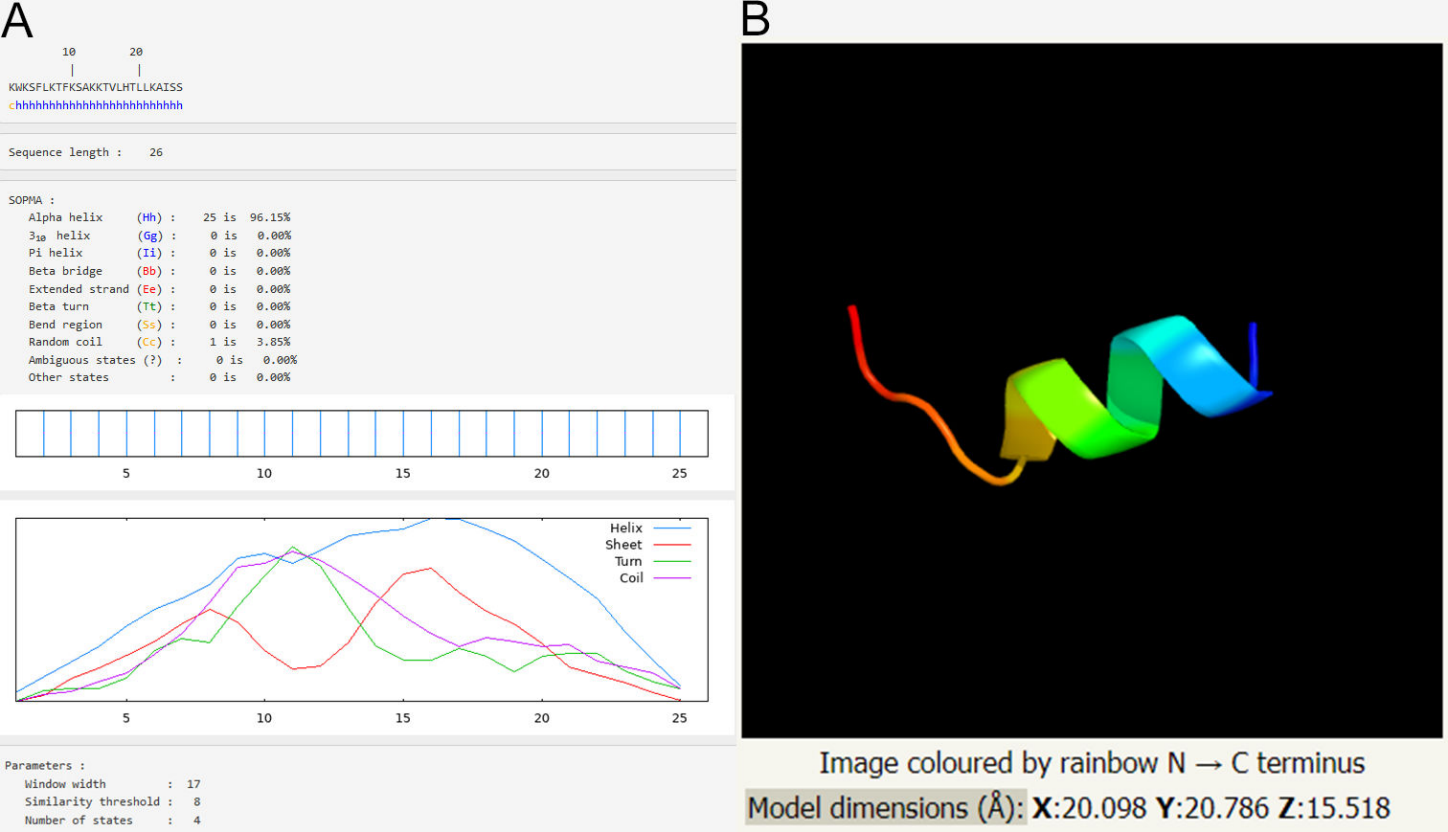
The secondary structure and 3D model of A20L. Expasy software predicted that the main structure of AMP A20L was α helix, which accounted for 96.15% (A). The 3D structure prediction of A20L by the Phyre2 software also shows an alpha helix structure (B).

As shown in Fig. 1, Expasy software predicted that the main structure of AMP A20L was α helix, and the content accounted for 96.15%. The 3D structure prediction of Phyre2 also showed an α helix structure (Fig. 1).

### The minimum inhibitory concentration (MIC) and minimum bactericidal concentration (MBC) of A20L against *K. pneumoniae*

The MIC and MBC of A20L and Meropenem (MEM) against 11 CRKP strains and 9 carbapenem-sensitive *Klebsiella pneumoniae* (CSKP) strains were shown in Table 2. The results showed that A20L had efficient antibacterial activity against CRKP and CSKP, with MIC values ranging from 4 to 8 μg/mL and MBC values ranging from 4 to >16 µg/mL.

**TABLE 2:** Antimicrobial susceptible test of *K. pneumoniae^a^*

Strains		MIC (μg/mL)		MBC (μg/mL)
		A20L	MEM	A20L
CRKP	FK4038	8	>128^R^	8
	FK4109	4	128^R^	4
	FK4111	4	32^R^	4
	FK7018	4	8^R^	4
	FK7779	4	4^R^	4
	FK7833	4	64^R^	4
	FK7836	4	8^R^	>16
	FK7861	4	>64^R^	4
	FK7921	8	64^R^	8
	FK7978	4	64^R^	>16
	FK8401	8	8^R^	8
CSKP	FK4859	4	0.25^S^	8
	FK4861	8	0.125^S^	8
	FK4864	4	0.125^S^	8
	FK4865	4	0.125^S^	4
	FK4867	4	0.125^S^	4
	FK4868	8	0.125^S^	8
	FK4869	4	0.125^S^	4
	FK6756	8	0.25^S^	8
Quality control strain	ATCC25922	4	0.03^S^	8

^*a*^ MEM, meropenem; MIC, the minimum inhibitory concentration; MBC, the minimum bactericidal concentration; Breakpoints (S-R): MEM (1–4 μg/mL); S-R represents the susceptible (S) breakpoint to resistant (R) breakpoint. The antimicrobial susceptible test of A20L was performed in Muller-Hinton Broth (MHB without Ca^2+^ and Mg^2+^.

### Scanning electron microscope image

**Fig 2:**
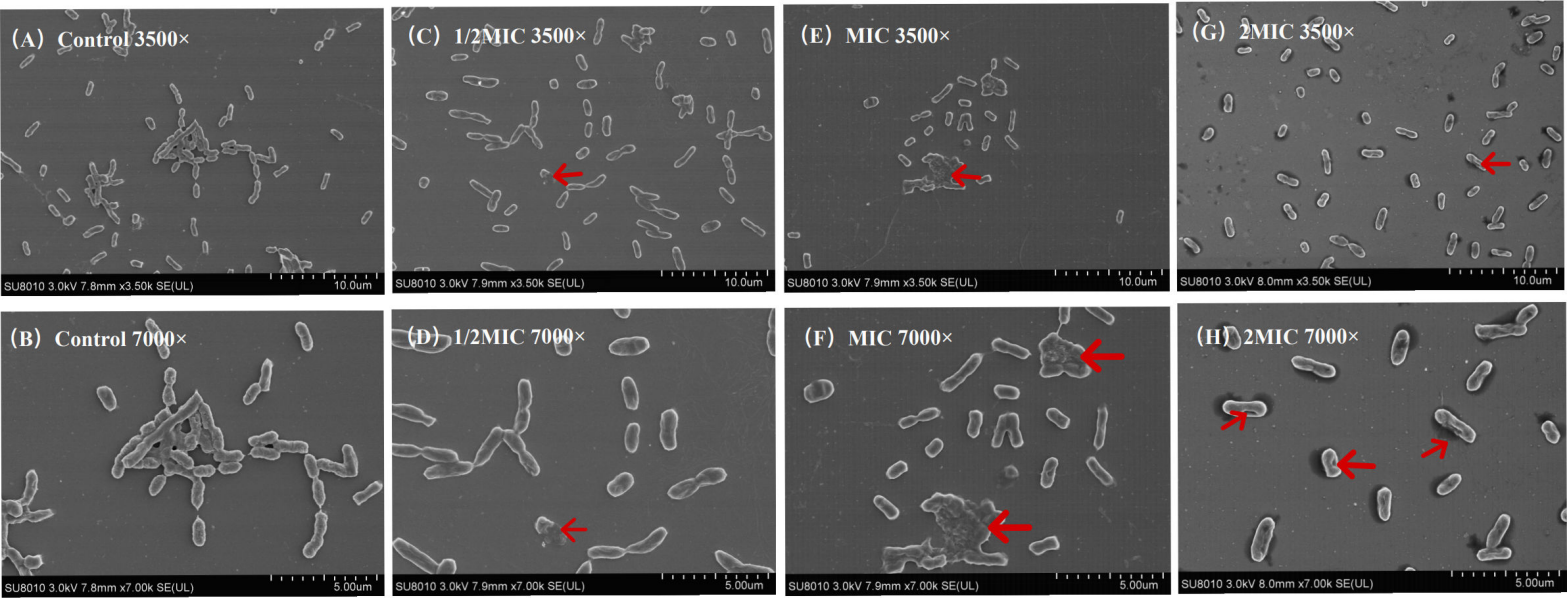
The effect of A20L on the morphology of *K. pneumoniae* FK6756 under SME. (A) LB broth control at 3000× magnification. (B) LB broth control at 7000× magnification. (C) A20L (1/2 MIC) at 3000× magnification. (D) A20L (1/2 MIC) at 7000× magnification. (E) A20L (MIC) at 3000× magnification. (F) A20L (MIC) at 7000× magnification. (G) A20L (2 MIC) at 3000× magnification. (H) A20L (2 MIC) at 7000× magnification.

The effect of A20L on bacterial morphology was further observed by scanning electron microscopy (SEM). As shown in Fig. 2, untreated *K. pneumoniae* was morphologically intact. However, the samples treated with 1/2 MIC, MIC, and 2 MIC concentrations of A20L showed changes in bacterial morphology, with FK6756 cell morphology crumpled, collapsed, and ruptured (red arrow).

### A20L improved the survival rate of *Galleria mellonella* larvae infected with *K. pneumoniae*

**Fig 3:**
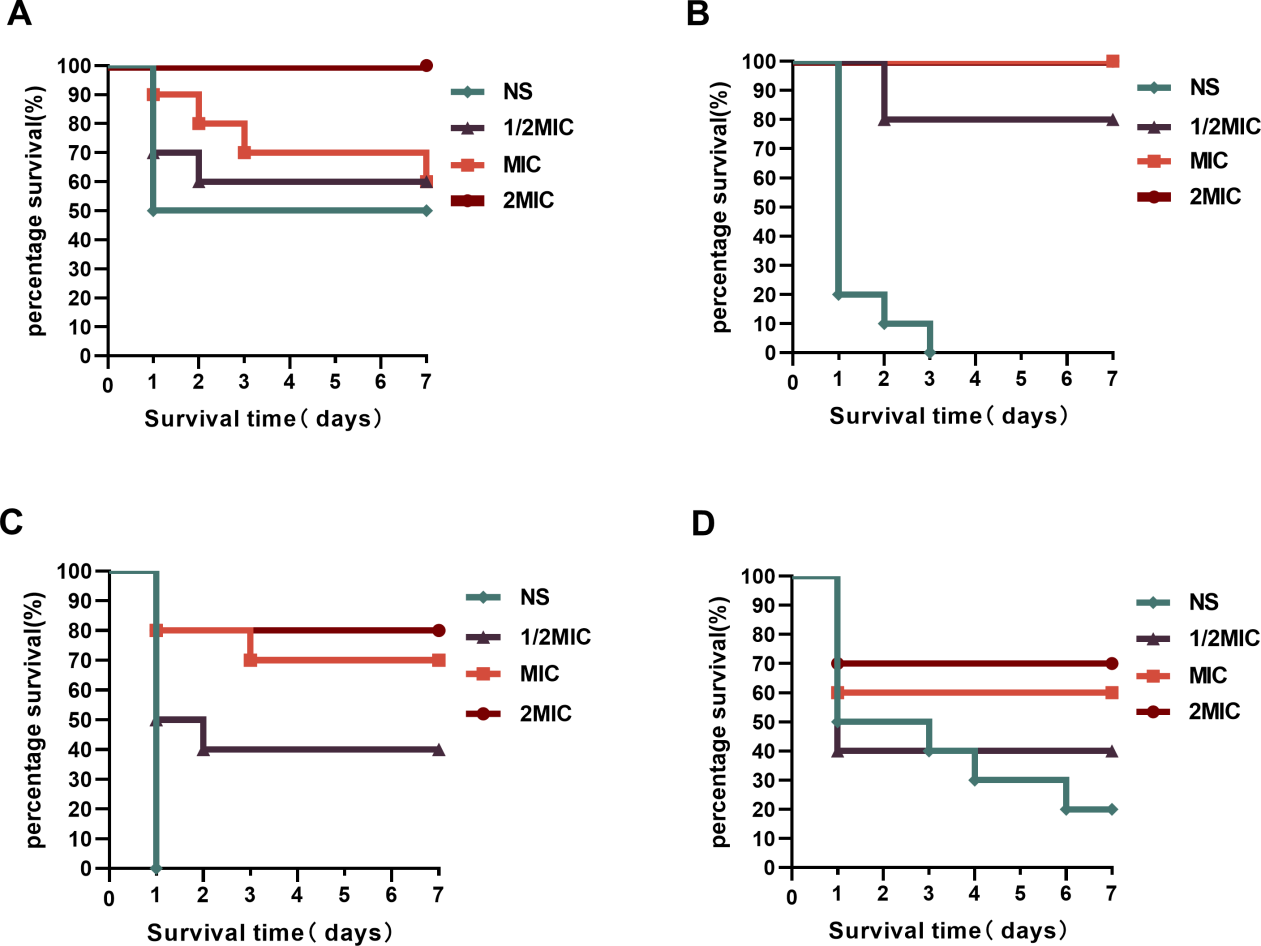
Effect of A20L treatment on survival of *G. mellonella* larvae infected with *K. pneumoniae*. (A) The survival rate of larvae infected with *K. pneumoniae* FK4861 after 7 days. (B) The survival rate of larvae infected with *K. pneumoniae* FK7921 after 7 days. In Fig. 3B, the curves of 2MIC and MIC overlap. (C) The survival rate of larvae infected with *K. pneumoniae* FK6756 after 7 days. (D) The survival rate of larvae infected with *K. pneumoniae* FK8401 after 7 days. Data were analyzed by log rank test.

To determine the therapeutic effect of A20L *in vivo*, *G. mellonella* survival assays were performed. According to the results shown in Fig. 3, different concentrations of A20L increased the survival of larvae infected with *K. pneumoniae*. The results showed that A20L has specific *in vivo* efficacy.

### Inhibition and eradication biofilm activity of A20L

**Fig 4:**
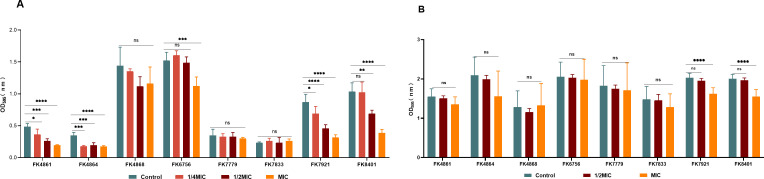
Antibiofilm activity of A20L against *K. pneumoniae*. (A) Biofilm inhibition effects of A20L on eight *K. pneumoniae* strains at different concentrations. (B) Biofilm eradication effects of A20L on eight *K. pneumoniae* strains at different concentrations. Data were analyzed by ANOVA; (ns, no statistically significant; *P < 0.05; **P < 0.005; ***P < 0.001; ****P < 0.0001). OD_595_, optical density at 595 nm.

We tested the ability of A20L to inhibit *K. pneumoniae* biofilm formation and damage the strain’s already-formed mature biofilm using the CV assays. As shown in Fig. 4A, A20L demonstrated biofilm suppression on half (4/8) of the tested strains at 1/2 MIC concentration. A20L with MIC concentration resulted in biofilm suppression for more than half (5/8) of the tested strains. Figure 4B showed that under MIC concentration, A20L had a removal effect on part of (2/8) mature biofilms of experimental strains.

### Safety of A20L *in vivo* and *in vitro*

**Fig 5:**
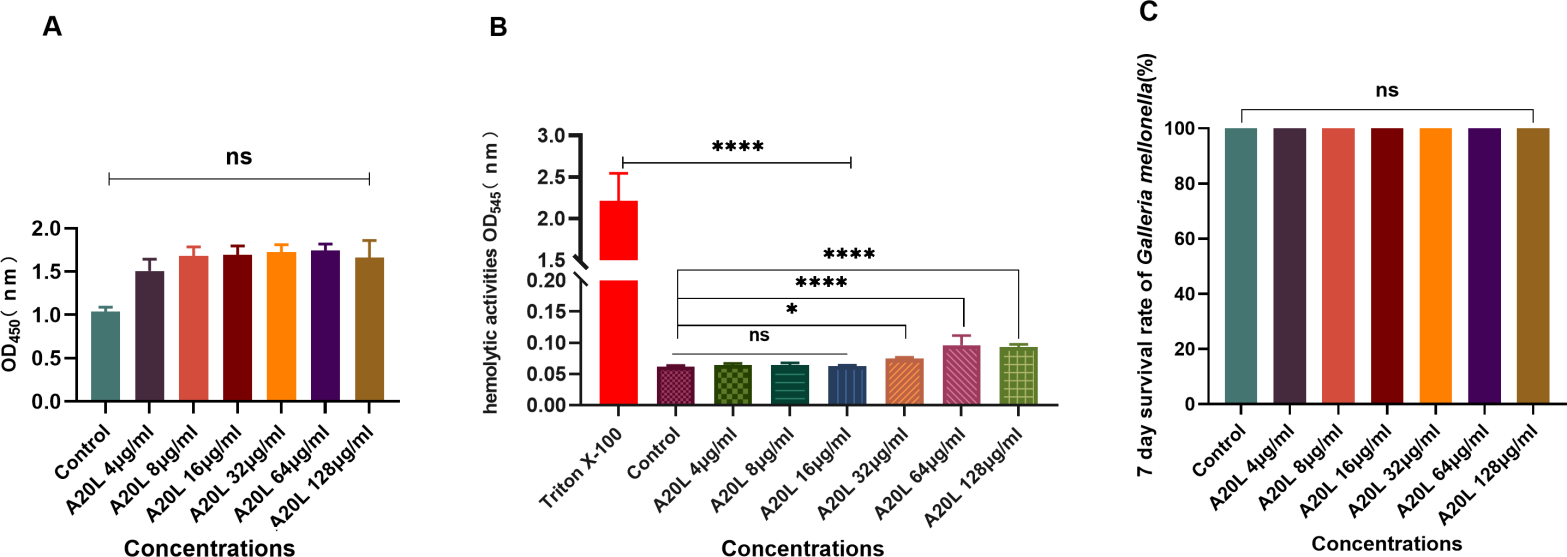
Safety assessment of A20L *in vitro* and *in vivo*. (A) CCK-8 assay was used to determine the cytotoxicity of different concentrations of A20L on RAW264.7 cells. (B) Hemolytic activity of different concentrations of A20L on mouse erythrocytes. (C) *In vivo* toxicity of different concentrations of A20L to *G. mellonella* larvae. Data were analyzed by ANOVA; (ns, no statistically significant; **P < 0.005; ***P < 0.001; ****P < 0.0001).

The cytotoxicity of various concentrations of A20L to RAW264.7 cells was detected by Cell Counting Kit-8 (CCK-8), and no cytotoxicity was observed when the concentration of A20L reached up to 128 µg/mL (Fig. 5A). As shown in Fig. 5B, A20L exhibited hemolytic activity on mouse erythrocytes at levels above 32 µg/mL. Fortunately, the concentrations we used were all lower than 32 µg/mL. The *in vivo* safety of A20L at different concentrations was investigated by *G. mellonella*. The results showed 100% of the larvae survived after 7 days being injected with up to 128 µg/mL of A20L (Fig. 5C). It was indicated that both *in vitro* and *in vivo* safety was achieved with the antimicrobial doses we utilized.

### Stability of A20L under different physicochemical conditions

We determined the effects of temperature, serum, and pair cations on the antibacterial activity of A20L. As shown in Table 3, the MICs of A20L did not change after 3 h of storage at different temperatures. After incubating with 5% FBS for 24 h, its antibacterial activity did not change. However, A20L was less resistant to 10% FBS and cations (Tables 4 and 5). The above results indicated that the antibacterial activity of A20L is well maintained at different temperatures, but it is less resistant to cations and 10% FBS.

**TABLE 3:** MIC of A20L after incubation at different temperatures^*a*^

Strains	MIC (μg/mL)						
	Before incubation	After incubation at					
		−80°C	−20°C	4°C	25°C	37°C	60°C
FK7779	4	4	4	4	4	4	4
FK7921	8	8	8	8	8	8	8
FK8401	8	8	8	8	8	8	8

^*a*^ MIC, Minimum inhibitory concentration. The dissolved peptides were treated at different temperatures (−80, −20, 4, 25, 37 and 60°C) for 3 h. The antimicrobial susceptible test of A20L was performed in MHB without Ca^2+^ and Mg^2+^.

**TABLE 4:** MIC of A20L after incubation in FBS^*a*^

Strains	MIC (μg/mL)		
	Before incubation	After incubation with	
		5% FBS	10% FBS
FK7779	4	4	32
FK7921	8	8	16
FK8401	8	8	32

^*a*^ MIC, Minimum inhibitory concentration; FBS, fetal bovine serum. The antimicrobial susceptible test of A20L was performed in MHB without Ca^2+^ and Mg^2+^. A20L was added to 5% and 10% FBS and incubated at 37°C for 24 h.

**TABLE 5:** MIC of A20L in the presence of cations

Strains	MIC^*a*^ (μg/mL)		
	Absence of cations	Presence of cations	
		Ca^2+^	Mg^2+^
FK7779	4	>128	>128
FK7921	8	>128	>128
FK8401	8	>128	>128

^*a*^ MIC, Minimum inhibitory concentration.

### A20L can increase membrane permeability of *K. pneumoniae*

**Fig 6:**
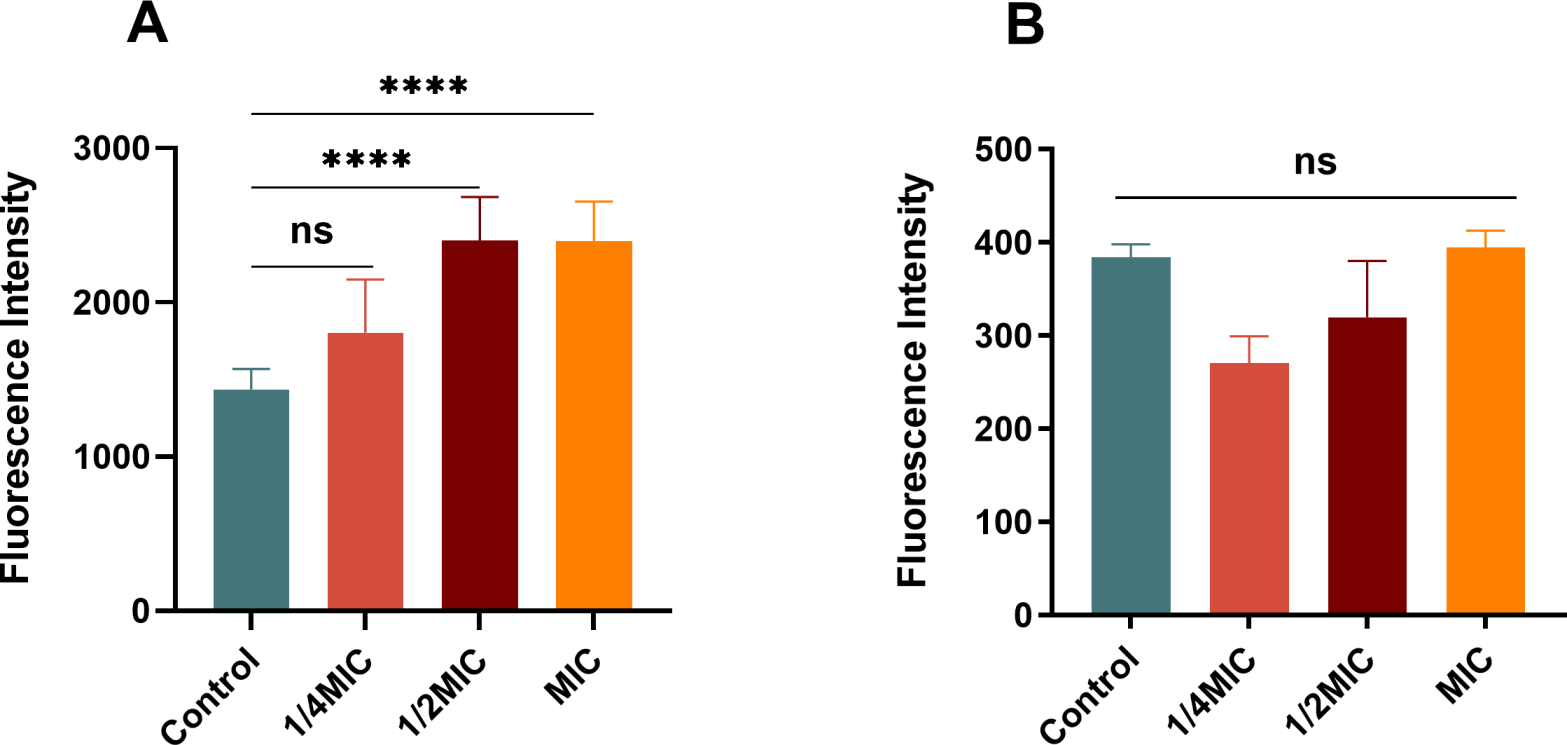
Cytoplasmic membrane permeability assays. Fluorescence intensities of NPN (A) and PI (B) after treatment with *K. pneumoniae* FK8401 with different concentrations of A20L. Data were analyzed by ANOVA; (ns, no statistically significant; ***P < 0.001; ****P < 0.0001).

The antimicrobial activity of A20L prompted us to wonder whether A20L kills bacteria via a membrane destruction mechanism. To determine the damaging effect of A20L on bacterial membranes, 1-N-phenylnaphthylamine (NPN) and propidium iodide (PI) staining experiments were performed. According to Fig. 6A, our results showed that A20L could increase the permeability of the outer membrane of *K. pneumoniae* FK8401, and the degree of the leakage increased with the increase of A20L concentration. At the same time, external toxic molecules might also be allowed to enter the cells. However, there was no obvious damage to the inner membrane of bacterial cells (Fig. 6B).

### A20L can inhibit the expression of inflammatory factors

**Fig 7:**
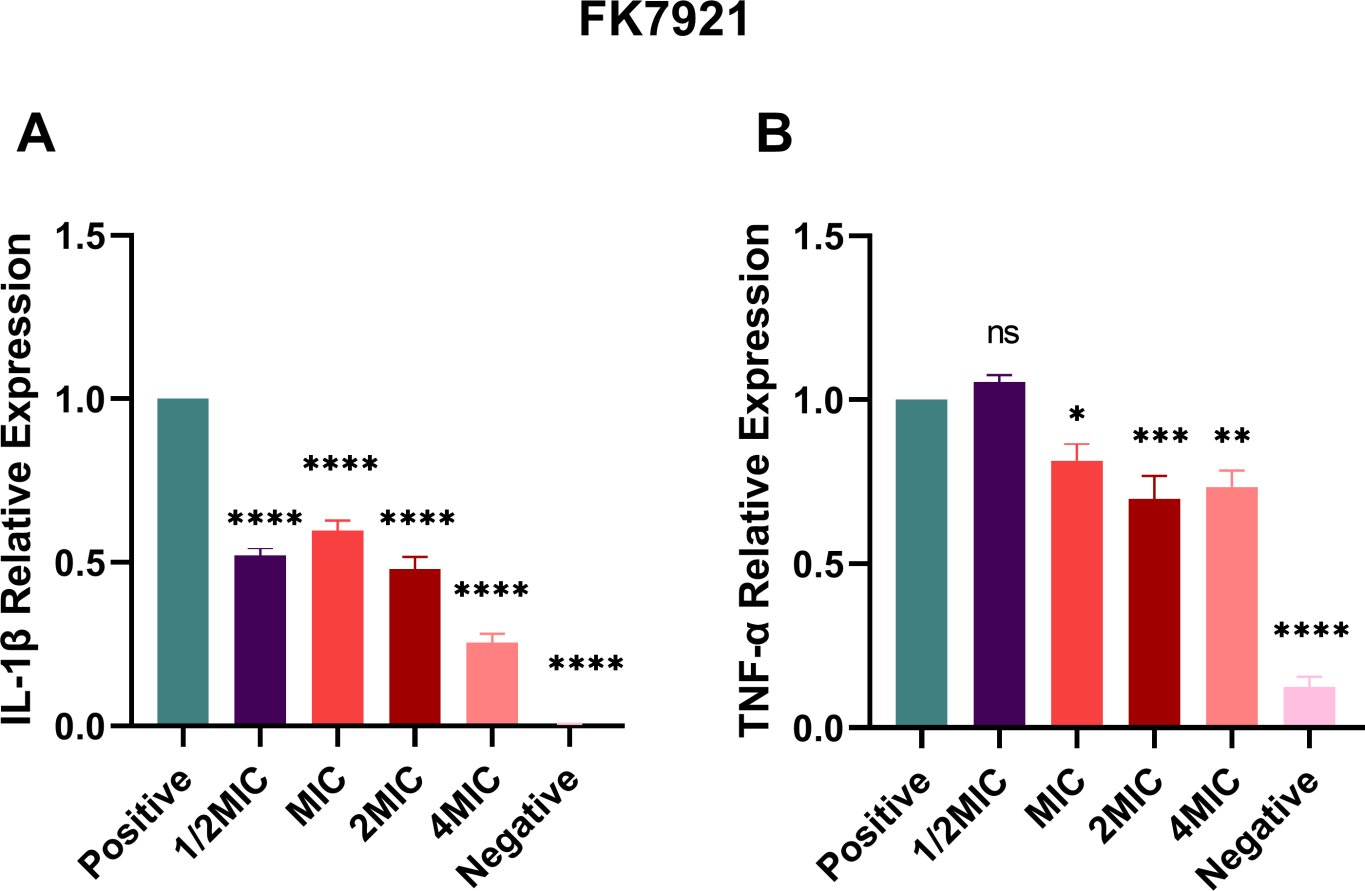
A20L inhibited the relative expression of inflammatory cytokines in RAW 264.7 cells infected with *K. pneumoniae* FK7921. Data were analyzed by ANOVA; (ns, not statistically significant; *P < 0.05; **P < 0.005; ***P < 0.001; ****P < 0.0001).

Compared with negative control, *K. pneumoniae* FK7921 significantly induced the expression of inflammatory cytokines IL-1β and TNF-α in RAW264.7 cells. A20L treatment inhibited the expression of inflammatory factors, demonstrating its anti-inflammatory activity (Fig. 7).

## DISCUSSION

CRKP is often resistant to numerous antibiotics, including fluoroquinolones, aminoglycosides, and β-lactams (18). Therefore, infections caused by these strains often face treatment failure. Several strategies are emerging to target bacterial resistance, such as the development of novel antimicrobial agents, phage endolysins, nanomaterials, and antimicrobial strategies for combination therapy (19–21).

AMPs, which have action against the invasion of foreign pathogens, are another novel antimicrobial agents recently suggested (22). AMPs characterized by short peptides, which generally include 100 or fewer amino acid residues (23), are thought to have a wide range of antibacterial action and little resistance potential, making them a viable replacement for conventional antibiotics (24). Most AMPs cause microbial death through non-specific interaction with the membrane surface and destruction of membrane integrity (25).

It has been demonstrated that A20L is an AMP with 26 amino acids that exhibits antibacterial, antifungal, and anticancer properties (26). However, its antibacterial activity against *K. pneumoniae* has not been comprehensively tested in previous studies. The aim of this study was to evaluate A20L’s *in vitro* and *in vivo* antibacterial, antibiofilm activity and potential mechanisms against *K. pneumoniae* clinical isolates, as well as its safety and stability.

In this study, we found that A20L exhibited potent antimicrobial activity against carbapenem-resistant and sensitive *K. pneumoniae* using microbroth dilution method. We also visually observed the effect of A20L on bacterial morphology under electron microscopy. The antibiofilm activity of A20L was investigated by CV staining, and the results showed that A20L exhibited inhibitory and scavenging effects on the biofilm of part of the isolates. This revealed variations in A20L’s influence on biofilm development in various strains. This may be due to differences in the ability to form biofilms among individual strains and the presence of their specific characteristics (27).

We looked at the anti-infective efficacy *in vivo* in addition to the antibacterial activity *in vitro*. We established a *G. mellonella in vivo* infection model and treated it with A20L. We found that A20L improved the survival rate of infected larvae to some extent, and thus corresponding efficacy *in vivo*.

The main antibacterial activity of AMPs is attributed to membrane solubilization mechanisms that directly interfere with the integrity of bacterial cell membranes and cell walls (28). Therefore, we used membrane permeability tests to verify and observe whether A20L exerted antibacterial activity through membrane destruction mechanisms. The results suggested that A20L might act on the outer membrane of bacterial cells.

In addition, inflammatory responses play an important role in bacterial infections, producing inflammatory cytokines. We observed that A20L inhibited the production of proinflammatory factors IL-1β and TNF-α by quantitative reverse transcription polymerase chain reaction (RT-qPCR). This result suggested that A20L inhibits inflammatory processes during bacterial infection.

The clinical development of AMPs has been limited by a variety of factors, including possible toxicity and stability (28, 29). We proved that A20L is secure through *in vitro* cell tests and *in vivo* toxicity tests using *G. mellonella*. Additionally, we examined the stability of A20L at various temperatures, serum concentrations, and in the presence of cations. The results showed that the antibacterial activity of A20L might be impacted by cations and high serum concentrations. Therefore, in order to develop novel AMPs, reducing toxicity and improving stability are breakthroughs that need to be made in future research.

In conclusion, this study reported that antibacterial peptide A20L has antibacterial and antibiofilm activity *in vitro* and anti-infective efficacy *in vivo* against *K. pneumoniae*, which may play a role through membrane destruction mechanism, and is stable and safe, making it a potential antibiotic substitute.

## MATERIALS AND METHODS

### Bacterial strains

In this study, 19 clinical *K. pneumoniae* strains were isolated from the First Affiliated Hospital of Wenzhou Medical University, including 11 CRKP and 8 CSKP. The clinical data of the patients and the characteristics of the analyzed strains are shown in Table S1. All isolates were identified by matrix-assisted laser desorption/ionization time-of-flight mass spectrometry (MALDI-TOF/MS; BioMerieux, Lyon, France). ATCC25922 was a quality control strain purchased from the National Clinical Laboratory Center. All tested strains were frozen in LB broth (Sigma-Aldrich, St. Louis, USA) with 30% (vol/vol) glycerin at −80℃ for future use.

### Peptides and chemicals

Referring to previous studies, the amino acid sequence of A20L (KWKSFLKTFKSAKKTVLHTLLKAISS) was obtained (17), and the customized peptide in the form of powder flocculus was synthesized by Nanjing TGpeptide Biotechnology Co., LTD (Nanjiang, China). Subsequently, the peptide was verified to match the previous peptides by high performance liquid chromatography (HPLC) and mass spectrometry. MEM was purchased from MedChemExpress Biotechnology Co., LTD (USA). Medium included MHB and Luria-Bertani (LB) agar were purchased from Thermo Fisher Scientific (USA). Phosphate buffered saline (PBS) and alkaline phosphatase kits were purchased from Solarbio Science & Technology Co., LTD (Beijing, China).

### Physicochemical properties and structural prediction of A20L

Expasy and Prabi software were used to predict and analyze the physicochemical properties of A20L. The analytical parameters included molecular weight of AMP A20L, amino acid residue, net charge, isoelectric point (PI), aliphatic index, hydrophilicity index (GRAVY), instability index, and alpha helix. Phyre2 software was used to predict the secondary structure and 3D model of A20L.

### Antimicrobial susceptibility tests (AST) for AMPs and antibiotics

The MIC and MBC of the peptide A20L were determined according to the broth microdilution method (BMD) recommended by the Clinical and Laboratory Standards Institute (CLSI 2022) with minor modifications. In brief, the *K. pneumoniae* strains were inoculated on Columbia blood agar plates and incubated in a constant temperature incubator at 37℃ for 16–18 h. Stock solutions of A20L and MEM were prepared to four times the maximum concentration required using autoclave solvent PBS and normal saline (NS), respectively, and then stock solutions were diluted in a 96-well plate along the H-A column by multiple dilution. MEM was used to distinguish carbapenem-resistant or susceptible strains. A single colony was picked from a fresh overnight blood culture dish with a sterile cotton swab, and the bacterial solution was adjusted to 0.5 McFarland standard with sterile NS. The bacterial solution was diluted 1:100 times with MHB to a concentration of 10^6^ colony forming units (CFUs)/mL. 100 µL bacterial solution was added into 96-well cell culture plates containing 100 µL A20L and MEM at different concentrations, and then incubated at 37°C for 18–24 h. The results were interpreted according to CLSI 2022 standard. ATCC25922 was used as a quality control strain. MIC is defined as the lowest concentration that is visible with naked eye to inhibit bacterial growth. The CFU counting method was used to determine the MBC of A20L against *K. pneumoniae* (30). 100 µL of bacterial suspension was taken from the corresponding wells of MIC, 2 MIC, 4 MIC, and 8 MIC and coated on LB agar plates. After incubating at 37°C for 18–24 h, the lowest concentration corresponding to no bacterial growth on the plate was determined as MBC.

### SEM observation

To visualize the morphological effects of A20L on *K. pneumoniae*, SEM was performed referring to previous studies with slight modifications (31). The randomly selected strain FK6756 grown overnight was adjusted to 0.5 McFarland standard with sterile NS. A20L final concentrations of 1/2 MIC, MIC, and 2 MIC were assigned to groups. LB broth without drugs served as a blank control. Sterile round silicon discs were placed in each group, and 100 µL of bacterial suspension was added to each group and incubated at 37°C for 18–24 h. The silicon chip was removed after bacteria were attached, cleaned with PBS, fixed in 2.5% glutaraldehyde at 4°C overnight, dehydrated with graded series (0%, 50%, 70%, 80%, 90%, 100%) ethanol for 10 minutes, and dried naturally. The chips were coated with platinum and were then observed under SEM (Hitachi SU8010, Japan).

### *In vivo* infection model of *G. Mellonella*

Like other insects, *G. mellonella* lacks an adaptive immune system, but its innate system shares many similarities with that of mammals. It includes a cellular response, in which blood cells (immune cells close to mammalian neutrophils) are the key players responsible for cellular events, as well as humoral responses with soluble effector molecules (32). As per its immune system, it is resistant to different microbes and is often used as models for studying microbial pathogens. Over the past few decades, *G. mellonella* has been widely used to evaluate virulence and the efficacy of antimicrobial treatments (33, 34).

We further evaluated the *in vivo* efficacy of A20L by developing an *in vivo* infection model of *G. mellonella* (35). FK4861 and FK7921 were selected as experimental strains, inoculated on Columbia blood agar plates, and incubated at 37°C for 18–24 h. A single colony in the logarithmic phase of growth on a blood culture dish was dipped with a sterile cotton swab, and the bacterial solution was adjusted to 0.5 McFarland standard with sterile NS. The median lethal concentrations of the four strains FK4861, FK7921, FK6756, and FK8401 were obtained by a pre-experiment, so as to determine the required bacterial injection concentrations (36). The FK4861 bacterial solution was diluted to 1 × 10^6^ CFU/ml with NS, FK7921 bacterial solution to 1 × 10^7^ CFU/mL, FK6756 bacterial solution to 1 × 10^6^ CFU/mL and FK8401 bacterial solution to 1 × 10^3^ CFU/mL. *G. mellonella* larvae weighing about 200 mg with similar activity were selected. Ten larvae were grouped together, and 10 µL of the prepared bacterial solution was aspirated with a microsyringe and injected into the *G. mellonella* larvae via the lower left foot. One hour after bacterial infection, 10 µL of different concentrations of A20L (1/2 MIC × 7, MIC × 7, 2 MIC × 7) were used for treatment, and 10 µL of sterile NS was injected into the control group. The larvae were incubated at 37°C in a constant temperature incubator. The survival rate of *G. mellonella* was recorded at 1, 2, 3, 4, 5, 6, and 7 days after infection and the survival curve was plotted. When the larva does not respond to external physical stimuli, it can be considered dead.

### Biofilm inhibition and eradication assay

In order to investigate whether A20L has antibiofilm activity, CV staining was used to determine its inhibition effect on biofilms and clearance effect on mature biofilms. In brief, for biofilm inhibition assay, eight *K. pneumoniae* strains were selected as test subjects and inoculated on Columbia blood agar plates then incubated at 37℃ for 18–24 h. After that, individual colonies in the logarithmic growth phase were picked with a sterile cotton swab, and the cultures were adjusted to 0.5 McFarland standard using sterile NS. The bacterial suspension was then diluted 1:100 in LB broth and finally distributed to 96-well culture plates containing different concentrations of A20L (1/4 MIC, 1/2 MIC, and MIC). 100 µL of bacterial suspension was added to each well, and wells without drug served as negative controls. After incubation at 37℃ for 20–24 h, the medium in the 96-well plate was discarded with a pipette. Subsequently, planktonic cells were removed by rinsing with NS 2–3 times and allowed to dry and fix. 150 µL of 1% CV dye (Beijing Solarbio Biotechnology Co., LTD) was added to each well and placed at 37℃ for 15 min. Each well was washed twice with NS. The bound CV was then dissolved in 150 µL absolute ethanol per well. A microplate reader was used to detect absorbance at 595 nm (Multiskan FC) (37).

Strains and culture conditions for mature biofilm removal experiments were identical to those used in biofilm formation inhibition experiments. Bacterial samples were adjusted to 0.5 McFarland standard with sterile NS and then diluted 1:100 in LB broth. 200 µL of bacterial suspension was added to each well and incubated at 37°C for 20–24 h. After discarding the planktonic cells, 200 µL of A20L with different concentrations (1/2 MIC and MIC) was added to each well. The medium in the 96-well plates was discarded after incubation at 37°C for 20–24 h. The plates were washed 2–3 times, dried, and then fixed. CV was added and left for 15 min. After staining, CV was removed and the wells were washed with NS. Subsequently, absolute ethanol was used to dissolve the previously mentioned bound CV. Absorbance was measured at 595 nm using a microplate reader (37).

### Safety evaluation

To determine whether A20L was safe at the concentrations used, *in vitro* cytotoxicity assays (38), erythrocyte hemolysis assays (39), and *in vivo G. mellonella* toxicity assays (40) were performed. Macrophage RAW264.7 cells were cultured in Dulbecco Modified Eagle Medium (DMEM) supplemented with 10% heat inactivated FBS and incubated at 37°C, 5% CO_2_. The cells were treated with trypsin to obtain individual cells. Approximately 100 µL cell suspension containing 1 × 10^5^ cells was implanted into each well of the 96-well microplate. 10 µL of A20L (4, 8, 16, 32, 64, and 128 µg/mL) was added into the inoculated 96-well plate and the group without reagent served as negative control. Then incubated in 5% CO_2_ incubator at 37°C for 12 h. After that, 10 µL CCK-8 (Dojindo Laboratories, Japan) was added to each well and incubated indoors in the dark for 1 h. The absorbance of each well at 450 nm was recorded using a microplate reader.

Hemolysis tests were performed using red blood cells isolated from the blood of mice. The red blood cells were washed three times with PBS to remove the serum and diluted to 6% (vol/vol) with PBS. The diluted red blood cells suspension was incubated with peptides (4, 8, 16, 32, 64, and 128 µg/mL) prepared in PBS at 37°C for 30 min, and the optical density of the centrifuged supernatant was determined at 545 nm using a microplate reader. PBS alone and 0.1% Triton X-100 were used as negative and positive controls, respectively.

For *in vivo* toxicity testing, *G. mellonella* larvae with body weight of about 200 mg and comparable activity was selected. A group of 10 larvae was set up, and 10 µL of the prepared peptide solution (4, 8, 16, 32, 64, and 128 µg/mL) was injected into the *G. mellonella* through the lower left foot of the larvae with a microsyringe. In the control group, 10 µL of sterile NS was injected into each subject. Different groups of *G. mellonella* were incubated in an incubator at 37°C. The larvae were continuously monitored for 7 days to note any deaths. Larvae can be considered dead when they do not respond to external physical stimuli.

### Temperature, serum, and cationic stability tests

Temperature, serum, and cation stability assays were performed using methods reported in previous studies (41, 42) with minor modifications to assess the impact of these conditions on the antimicrobial activity of A20L. The dissolved peptides were treated at different temperatures (−80, −20, 4, 25, 37, and 60°C) for 3 h. Another group of peptides were added to 5% and 10% FBS and incubated at 37°C for 24 h. And the third group of peptides were treated with 25 µg/mL Ca^2+^ and 12.5 µg/mL Mg^2+^. Three strains were randomly selected to determine the MIC of the treated A20L.

### Membrane permeability assay

The effect of A20L on the envelope integrity of *K. pneumoniae* was detected by PI and NPN, as previously described (43), with slight modifications. Randomly selected *K. pneumoniae* FK8401 exponential phase collections were adjusted to a final concentration of 1 × 10^8^ CFU/mL. The bacterial solution was centrifuged at 5,000× *g* for 3 min, the supernatant was discarded, then cell pellets were resuspended in PBS, and this process was repeated 2–3 times. Dissolved peptides (1/4 MIC, 1/2 MIC, MIC) were added to each tube of bacterial solution, and tubes without peptides served as controls. After 2 h of shaking culture at 37°C, 1 mL of the solution was removed and the supernatant was discarded by centrifugation. Add 1 mL of prepared NPN (30 µg/mL) or PI (50 µg/mL) staining solution and incubate at 37°C for 20 min. It was then centrifuged and washed with PBS as described above and repeated twice to wash off the excess dye solution. PBS was added and mixed, and the fluorescence of NPN (excitation wavelength 350 nm, emission wavelength 420 nm) and PI (excitation wavelength 535 nm, emission wavelength 615 nm) was detected by a multifunctional microplate reader.

### Anti-inflammatory effect of A20L

We explored the effect of A20L on proinflammatory cytokines with slight modifications referring to previous studies (44). RAW 264.7 cells frozen in liquid nitrogen at −196°C were resuscitation and subcultured in DMEM containing 10% FBS and incubated at 37°C, 5% CO_2_. The RAW 264.7 cells (1 × 10^6^ cells well) were incubated overnight to attach to the wall.

The medium in the wells was discarded, and each well was washed three times with PBS. The cells were treated with *K. pneumoniae* FK7921 with a multiplicity of infection (MOI) of 10 and different concentrations of A20L for 2 h. Wells containing only bacteria served as positive controls, and wells containing no A20L or bacteria served as negative controls.

The total RNA of cells was extracted with RNA extraction kit (Beijing Kangwei Century Biotechnology Co., LTD), RNA quality was determined with A260/280, and cDNA was transcribed with PrimeScript RT kit (Beijing Kangwei Century Biotechnology Co., LTD) according to the manufacturer’s instructions. The cycle threshold (CT) values of IL-1β and tumor necrosis factor (TNF)-α were detected by TB GreenPremix Ex TaqTM II. The relative expression of IL-1β and TNF-α was analyzed by 2^−△△CT^ using β-actin as the internal gene. Primers used in this study are listed in Table S2.

### Statistical analysis

All experiments were performed triplicate. Data were expressed as the mean standard deviation of at least three independent experiments. One-way ANOVA, Log Rank test, and Student’s *t*-test were used for statistical analysis (ns, no statistically significant; *P < 0.05; **P < 0.005; ***P < 0.001; ****P < 0.0001). All statistical calculations were processed using GraphPad Prism 8.0 statistical software.

## SUPPLEMENTAL MATERIAL

Table S1 spectrum.03979-23-s0001.doc Patient's clinical data and characteristics of analyzed strains.

Table S2 spectrum.03979-23-s0002.doc qPCR Primer sequence, production size.

ASM does not own the copyrights to Supplemental Material that may be linked to, or accessed through, an article. The authors have granted ASM a non-exclusive, world-wide license to publish the Supplemental Material files. Please contact the corresponding author directly for reuse.
