# Supplementary material for: Antimicrobial peptide A20L: in vitro and in vivo antibacterial and antibiofilm activity against carbapenem-resistant Klebsiella pneumoniae
Source: Microbiol Spectr. 2024 Jul 9;12(8):e03979-23. doi: 10.1128/spectrum.03979-23 (PMC11302274; doi:10.1128/spectrum.03979-23)
Supplement: Table S1 — Patient's clinical data and characteristics of analyzed strains. [file spectrum.03979-23-s0001.doc]

TABLE S1 Patient’s clinical data and characteristics of analyzed strains.

| **Strains** | | **Isolation date** | **Age** | **Gender** | **Sample** | **Ward** |
| --- | --- | --- | --- | --- | --- | --- |
| CRKP | FK4038 | 02/06/2017 | 69 | M | Sputum | ICU |
|  | FK4109 | 02/06/2017 | 69 | M | Wound | ICU |
|  | FK4111 | 02/06/2017 | 69 | M | Feces | ICU |
|  | FK7018 | 14/07/2019 | 39 | M | Sputum | ICU |
|  | FK7779 | 25/12/2019 | 69 | M | Sputum | ICU |
|  | FK7833 | 11/01/2019 | 50 | M | Sputum | ICU |
|  | FK7836 | 10/01/2020 | 74 | M | Feces | ICU |
|  | FK7861 | 16/01/2020 | 42 | M | Feces | ICU |
|  | FK7921 | 06/02/2020 | 42 | M | Urine | ICU |
|  | FK7978 | 04/03/2020 | 65 | M | Sputum | Neurosurgery |
|  | FK8401 | 23/06/2020 | 54 | M | Sputum | ICU |
| CSKP | FK4859 | 01/02/2018 | 74 | F | Sputum | Neurosurgery |
|  | FK4861 | 31/01/2018 | 91 | M | Wound | Gastroenterology |
|  | FK4864 | 01/02/2018 | 68 | F | Blood | Infectious Department |
|  | FK4865 | 03/02/2018 | 59 | M | Pus | Otolaryngology |
|  | FK4867 | 01/02/2018 | 68 | M | Pus | Hepatobiliary Surgery |
|  | FK4868 | 02/02/2018 | 60 | M | Ascites | Hepatobiliary Surgery |
|  | FK4869 | 03/02/2018 | 50 | F | Drainage | Gynecology |
|  | FK6756 | 22/05/2019 | 47 | M | Urine | Emergency Department |

Abbreviations: CRKP, carbapenem-resistant *Klebsiella pneumoniae*; CSKP, carbapenem-sensitive *Klebsiella pneumoniae.* ICU, Intensive Care Unit. M, male; F , female.
