# Supplementary material for: Antimicrobial peptide A20L: in vitro and in vivo antibacterial and antibiofilm activity against carbapenem-resistant Klebsiella pneumoniae
Source: Microbiol Spectr. 2024 Jul 9;12(8):e03979-23. doi: 10.1128/spectrum.03979-23 (PMC11302274; doi:10.1128/spectrum.03979-23)
Supplement: Table S2 — qPCR Primer sequence, production size. [file spectrum.03979-23-s0002.doc]

**Table S2.** qPCR Primer sequence, production size.

| **Primer** | **Sequence** | **Size(bp)** | |
| --- | --- | --- | --- |
| IL-1β | **F:** **GAAATGCCACCTTTTGACAGTG**  **R:** TGGATGCTCTCATCAGGACAG | | 116 |
| TNF-α | F: ATGAGAAGTTCCCAAATGGC  **R: CTCCACTTGGTGGTTTGCTA** | | 125 |
| β-actin | **F:**GGAGATTACTGCCCTGGCTCCTA  **R:** **GACTCATCGTACTCCTGCTTGCTG** | | 150 |
